# Reducing overdose deaths among persons with opioid use disorder in connecticut

**DOI:** 10.1186/s12954-024-01026-6

**Published:** 2024-05-28

**Authors:** Joy D. Scheidell, Tarlise N. Townsend, Qinlian Zhou, Prima Manandhar-Sasaki, Ramon Rodriguez-Santana, Mark Jenkins, Marianne Buchelli, Dyanna L. Charles, Jillian M. Frechette, Jasmine I-Shin Su, R. Scott Braithwaite

**Affiliations:** 1 Department of Health Sciences, University of Central Florida, PO Box 160000, Orlando, FL 32816 USA; 2 Department of Population Health, New York University Grossman School of Medicine, 227 E. 30th St, New York, NY 10016 USA; 3 Center for Opioid Epidemiology and Policy, New York University Grossman School of Medicine, New York, NY USA; 4 HIV Prevention Program, Connecticut Department of Public Health, 410 Capitol Avenue, MS #11APV, Hartford, CT 06134-0308 USA; 5 TB, HIV, STD and Viral Hepatitis Section, Connecticut Department of Public Health, 410 Capitol Avenue, MS #11APV, Hartford, CT 06134 USA; 6 Connecticut Harm Reduction Alliance, 28 Grand St, Hartford, CT 06106 USA

**Keywords:** Opioid use disorder, Opiate overdose, Harm reduction, Opiate medication-assisted treatment, Cost-effectiveness analysis, Modeling

## Abstract

**Background:**

People in Connecticut are now more likely to die of a drug-related overdose than a traffic accident. While Connecticut has had some success in slowing the rise in overdose death rates, substantial additional progress is necessary.

**Methods:**

We developed, verified, and calibrated a mechanistic simulation of alternative overdose prevention policy options, including scaling up naloxone (NLX) distribution in the community and medications for opioid use disorder (OUD) among people who are incarcerated (MOUD-INC) and in the community (MOUD-COM) in a simulated cohort of people with OUD in Connecticut. We estimated how maximally scaling up each option individually and in combinations would impact 5-year overdose deaths, life-years, and quality-adjusted life-years. All costs were assessed in 2021 USD, employing a health sector perspective in base-case analyses and a societal perspective in sensitivity analyses, using a 3% discount rate and 5-year and lifetime time horizons.

**Results:**

Maximally scaling NLX alone reduces overdose deaths 20% in the next 5 years at a favorable incremental cost-effectiveness ratio (ICER); if injectable rather than intranasal NLX was distributed, 240 additional overdose deaths could be prevented. Maximally scaling MOUD-COM and MOUD-INC alone reduce overdose deaths by 14% and 6% respectively at favorable ICERS. Considering all permutations of scaling up policies, scaling NLX and MOUD-COM together is the cost-effective choice, reducing overdose deaths 32% at ICER $19,000/QALY. In sensitivity analyses using a societal perspective, all policy options were cost saving and overdose deaths reduced 33% over 5 years while saving society $338,000 per capita over the simulated cohort lifetime.

**Conclusions:**

Maximally scaling access to naloxone and MOUD in the community can reduce 5-year overdose deaths by 32% among people with OUD in Connecticut under realistic budget scenarios. If societal cost savings due to increased productivity and reduced crime costs are considered, one-third of overdose deaths can be reduced by maximally scaling all three policy options, while saving money.

**Supplementary Information:**

The online version contains supplementary material available at 10.1186/s12954-024-01026-6.

## Introduction

The United States (US) continues to struggle to curtail the opioid overdose crisis. The crisis has evolved from early waves featuring prescription opioids and heroin to the current wave of illicitly manufactured synthetic opioids including fentanyl [1]. In 2021, over 100,000 people died from a drug-involved overdose, which is a 14% increase from 2020, and opioids were involved in 75% of those overdose deaths [2]. To combat the worsening crisis, enhanced approaches to overdose interventions are necessary.

Distribution of naloxone (NLX) for overdose reversal has consistently been found to be cost effective [3–10], particularly when targeted to laypeople who are likely to witness or experience overdose [11]. During an overdose, when a matter of minutes may make the difference between survival and fatality, laypeople are often the true first responders by administering NLX before or while seeking professional medical attention. In addition, simulation models suggest that targeting community-based NLX distribution to people who use illicit opioids, including people who inject drugs (PWID) and the sites that they may frequent (e.g., syringe services programs) could significantly reduce overdose deaths, increase life expectancy, and be highly cost effective [7, 8].

While the cost effectiveness of distributing NLX is well established, less is known about the optimal combination of NLX formulations. Formulations currently available include an injectable naloxone formulations used for intravenous, intramuscular, or subcutaneous administration (INF), and a non-injectable version used for intranasal administration (IN) [12]. The injectable formulation has the advantage of reduced expense [13], but the disadvantage of lower acceptability to laypersons who are uncomfortable with injections, although this disadvantage may not apply to PWID and others without aversion to injections [14]. While comparative effectiveness research has demonstrated that intranasal NLX is as effective as injectable NLX for managing opioid overdose in pre-hospital settings [15] and economic evaluations indicate NLX distribution is cost effective [16], there is sparse research comparing the cost effectiveness of the two formulations.

Along with NLX distribution, treatment of opioid use disorder (OUD) is a vital component of overdose prevention and reduction strategies. People who misuse prescription opioids and/or use illicit opioids such as heroin may progress to OUD, which is characterized by regular use of opioids and experience of physical dependence (e.g., increased tolerance, withdrawal), loss of control (e.g., inability to quit), and consequences (e.g., interference with responsibilities) [17]. People with OUD are at increased risk of mortality due to overdose [18, 19], and treatment with medications for OUD (MOUD) such as methadone and buprenorphine greatly reduces overdose risk [20–22]. People who have been incarcerated have elevated rates of OUD and overdose compared to the general population [23], and periods of detainment often offer the opportunity to initiate treatment with MOUD [24]. People with OUD frequently cycle in and out of carceral settings and when they return to the community, their rates of overdose mortality drastically increase [25, 26]. Hence, treatment capacity both in the community and in carceral settings are key aspects of a public health strategy to reduce overdose mortality.

While modeling studies have identified cost-effective overdose prevention strategies [27, 28], it is unclear how resources should be allocated across these strategies to maximally avert overdoses given budget constraints. In the present study, we evaluate the potential for strategies to avert overdoses cost effectively using Connecticut as a case study. Connecticut has seen marked rises in overdose mortality and is among the US states with the highest overdose rates [29], with death from overdose more likely to occur than motor vehicle deaths [30]. We assessed the amount of benefit for the money spent (i.e., cost effectiveness) and number of overdose deaths prevented among people with OUD in Connecticut (CT) that could be achieved through NLX distribution and MOUD in community and carceral settings, both independently and in combination.

## Methods

### Model structure

The research aims, model structure, and parameters were guided through contributions by key stakeholders in the CT Department of Public Health (DPH) and CT Department of Correction (DOC). We used a probabilistic Markov model to simulate a hypothetical cohort of people with OUD in CT to compare the impact of the strategies on life expectancy, quality-adjusted life-years (QALY), number and percentage of opioid overdose deaths prevented over 5 years and the cohort’s lifetime, and total costs. We calculated the cost-effectiveness of each strategy using the incremental cost-effectiveness ratio (ICER), which represents the incremental change in costs divided by the incremental change in benefits versus the next-best intervention, measured in costs per QALY. The frontier represents the most cost-effective strategies at varying willingness-to-pay thresholds; the default willingness-to-pay is $100,000 per QALY based upon prior literature [31].

The model simulates a cohort of hypothetical individuals with OUD in CT. Each hypothetical person in the simulated cohort begins with a starting age, sex (i.e., male, female) and opioid use state (e.g., on treatment, in remission). Simulated individuals then go through a virtual life in the model that consists of many cycles, with the length of a cycle set to one day. In each cycle, individuals in the simulated cohort age by one day, potentially transition between incarceration and the community, potentially change OUD statuses, potentially experience an overdose event that may or may not be fatal, and potentially die from other causes. If an individual does not die of any cause in a cycle, the cycles continue. Based on NSDUH report [32] and adjustments suggested by Keyes et al. [33], the simulated cohort consisted of 90,895 people with OUD in CT, among which 3% are incarcerated at beginning [32, 34] and the rest start in the community (14% of whom have prior incarceration history) [35]. The technical appendix describes in detail how the simulations were conducted.

**Fig. 1:**
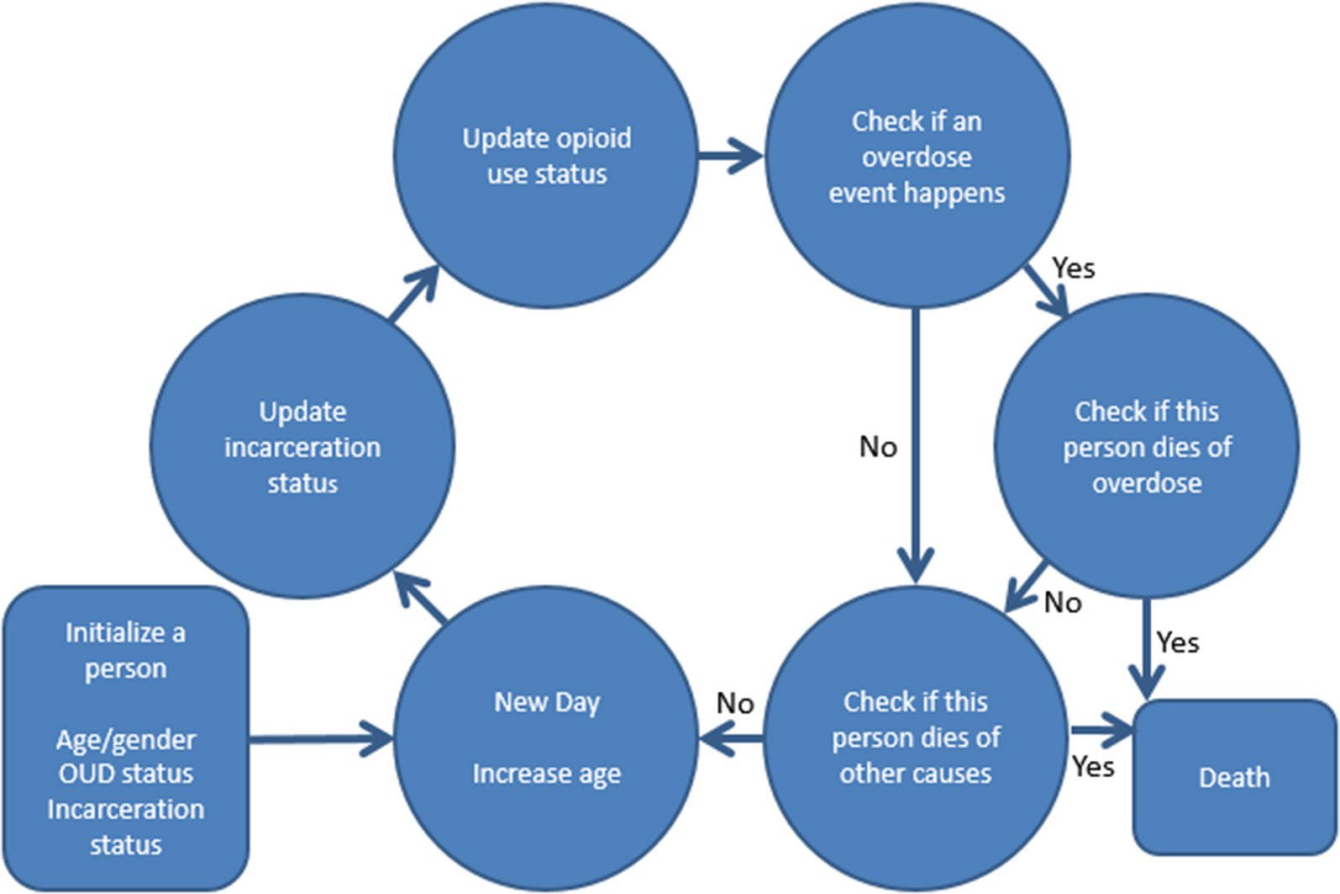
Algorithmic flowchart for a simulated person

Figure 1 illustrates the conceptual structure of the model. Individuals may be in the community or incarcerated. While incarcerated, they can be in two states: on treatment (+MOUD +remission), or not on treatment (−MOUD +remission). We made simplifying assumptions that: (1) few people who are incarcerated access non-prescription opioids and are therefore considered +remission, and (2) transitions between these two states are rare. Upon release from incarceration, individuals can transition between three states: *on treatment* (+MOUD +remission or +MOUD−remission), *using opioids without treatment* (−MOUD−remission), or *not using opioids without treatment* (−MOUD +remission). The model enables specification of varying levels of community availability of MOUD (MOUD-COM), with higher levels of availability increasing the chances that an individual enters the +MOUD state. For those using non-prescription and/or illicit opioids (−MOUD−remission or +MOUD−remission), overdoses are possible and can be fatal. The model assumes that increasing NLX distribution in the community does not impact overdose rates but decreases the probability that an overdose is fatal. Individuals can move between the community and incarceration and may die of causes other than overdose in both locations.

We defined “overdose” as the rapid onset of loss of consciousness from which arousal was difficult or impossible after ingestion of substances. Overdose rates were assumed to be higher for people who inject drugs (PWID) and for people with prior overdose history (Table 1). People with OUD on treatment (i.e., +MOUD−remission) can still experience an overdose in the model, but their overdose risk is 38% lower compared to people with OUD who are not on treatment (i.e., −MOUD−remission) based on Larochelle 2018 [36]. Depending on various factors (e.g., overdose being witnessed, NLX availability), the probability of an overdose being fatal is 0.1–10% (Table 1; Additional file 1: Appendix). In addition to overdose deaths, simulated persons could die of non-overdose related causes in accord with age-specific mortality rates that were derived from US life tables (Additional file 1: Appendix). We also added a non-overdose excess mortality among people with OUD (annual rate of 0.010 if out of treatment and not in remission, 0.003 in treatment or remission) [37].

**Table 1:** Model parameters

Parameter	Base case	Range	Source
*Population*			
	Number		
People with opioid use disorder (POUD) in Connecticut	90,895	N/A	Krawczyk et al. 2022 [32]
Incarcerated POUD (baseline)	2748	N/A	Ferguson et al. 2019 [34]
	Proportion		
Previously incarcerated	0.14	N/A	Winkelman et al. 2018 [35], Krawczyk et al. 2022 [32]
Had a prior overdose	0.31	0.27–0.35	
People who inject drugs			Heimer et al. 2014 [38]
Among never incarcerated	0.05	0.04–0.06	Expert opinion (CTDOC) + calibration adjustment
Among ever incarcerated	0.3	0.24–0.36	Expert opinion (CTDOC)
Baseline MOUD in community			Expert opinion (CTDOC)
+MOUD ± remission	0.17	0.1–0.4	Expert opinion (CTDOC)
−MOUD −remission	0.63	Complement	
−MOUD +remission^a^	0.2	0.13–0.43	Expert opinion (CTDPH)
Baseline naloxone access in community			
Own naloxone in community	0.03	0.02–0.04	Freeman 2018 [39]
Distribution of naloxone kits in community			Freeman 2018
Intranasal	0.8	Complement	
Intramuscular	0.2	0.16–0.24	
Transition rates (annual)^b^			
*Community-to-community (baseline relapse risk period/* > *1 mo post-release)*			
+MOUD +remission → −MOUD −remission^c^	0.13–0.33^e^		
Rate ratio for relapse during 1-month post-release	10		
+MOUD +remission → −MOUD +remission	0.16–0.31^f^		See appendix for multiple references
−MOUD +remission → −MOUD −remission	0.01	0.01–0.15	
−MOUD +remission → +MOUD +remission	0.29	0.07–0.72	
−MOUD −remission → +MOUD +remission ^d^	0.34	0.12–0.35	
−MOUD −remission → −MOUD +remission	1.07	0.17–1.16	
−MOUD −remission → overdose			
PWID no prior overdose	1.8	1.4–2.2	Coffin 2013 (For high propensity to relapse group, +MOUD −remission + →MOUD +remission, use same values)
PWID with prior overdose	5.6	2.8–6.7	Coffin 2013
Non-PWID no prior overdose	0.03	0.02–0.04	Coffin 2013
Non-PWID with prior overdose	0.11	0.09–0.13	Coffin 2013
*Naloxone acquisition in community (annual)*			
In an SSP	0.51		Expert opinion (CTHRA) & CT DMHAS
Non-SSP	0.1		
Probabilities (one-time)			
*Community-to-incarceration*			
+MOUD +remission → −MOUD +remission ^g^	0.57		See appendix for multiple references
+MOUD +remission → +MOUD +remission ^h^	0.43	[N/A exogeneous policy setting]	
−MOUD +remission → +MOUD +remission	0.01		
−MOUD +remission → −MOUD +remission	0.99		
−MOUD −remission → +MOUD +remission	0.3		
−MOUD −remission → −MOUD +remission	0.7		
*Incarceration (SI or LI)-to-community*			
+MOUD +remission → −MOUD +remission	0.17–0.21^j^	0.06–0.3	See appendix for multiple references
+MOUD +remission → −MOUD −remission	0.30–0.38^k^	Complement	
+MOUD +remission → −MOUD −remission	0.40–0.53^l^	0.2–0.73	
−MOUD +remission → −MOUD +remission	0.15	Complement	
−MOUD +remission → −MOUD −remission	0.54	0.21–0.41	
−MOUD +remission → +MOUD +remission ^i^	0.31		
Overdose → overdose death (without naloxone or EMS)	0.10	0.06–0.22	
Overdose being witnessed			
PWID	0.79	0.55–0.90	
Non-PWID	0.79	0.55–0.90	
EMS called	0.6	0.58–0.62	
Intervention effects^m^			
MOUD on OD prevention, community concurrent users^n^,^o^	RR 0.62	0.41–0.92	Larochelle et al. 2018[36]
Naloxone on ODD prevention	RR 0.92	0.8–0.97	Coffin 2013[8]
Emergency medical services called on ODD prevention	RR 0.92	0.8–0.97	Coffin 2013 [8]
Utilities			
−MOUD +remission	0.82	0.67–0.97	Rhee 2019 [40]
Decrement in utility due to –MOUD −remission	0.09	0–0.38	Rhee 2019
Decrement in utility due to Incarceration	0.06	0–0.18	Chong 2009 [41]
Cost (2021 USD)			
Incarceration, annual	$42,837	28,558–64,256	CTDOC
Naloxone, per dose			
IM	$15	Oct-23	Rosenberg 2018 [42]
IN	$60	40–90	
MOUD, annual			
Incarceration	$7630^r^	5081–51017	CTDOC [cost table]
Community	$4099^s^	1396–29,186	Expert opinion, Murphy 2019, [43] Clemans-Cope 2020 [44]
Crime, annual^p^	$68,302	45,535–102,453	Krebs 2016 [45]
Productivity loss, annual ^q^	$32,427	21,618–48,641	BLS 2023[46]/expert opinion
Emergency medical services dispatch	$1638	1092–2457	Larimer 2009 [47]
Admittance to emergency department	$4652	3101–6978	Mallow 2018 [48]

^a^Asymptotes towards 50% above age 50

^b^In the community, only those who have a low propensity for relapse can transition to three states (−MOUD +remission, +MOUD +remission, −MOUD −remission). Those who have a high propensity for relapse can transition between two states only (−MOUD −remission, +MOUD −remission)

^c^For high propensity to relapse group, +MOUD −remission → −MOUD −remission, use same values

^d^For high propensity to relapse group, −MOUD -remission → +MOUD –remission, use same values

^e^See appendix for MOUD type-specific values

^f^See appendix for MOUD type-specific values

^g^For high propensity to relapse group, +MOUD −remission → −MOUD +remission, use same values

^h^For high propensity to relapse group, +MOUD −remission → +MOUD +remission, use same values

^i^For high propensity to relapse group, −MOUD +remission → +MOUD −remission, use same values

^j^See appendix for MOUD type-specific values

^k^See appendix for MOUD type-specific values

^l^See appendix for MOUD type-specific values

^m^RR: risk ratio

^n^Methadone includes 4-week induction period (Sordo et al 2017)

^o^Applied to the “−MOUD −remission → overdose” rates

^p^Applied to −MOUD remission individuals only

^q^Applied to incarcerated or −MOUD −remission individuals only

^r^Weighted average of methadone, buprenorphine-naloxone, XR-naltrexone and XR-buprenorphine. See appendix for MOUD type-specific values

^s^Weighted average of methadone, buprenorphine-naloxone, XR-naltrexone and XR-buprenorphine. See appendix for MOUD type-specific values

We assumed 30% of individuals with OUD who have ever been incarcerated are PWID, and 5% of individuals who have never been incarcerated are PWID, based on expert estimation. Compared to those who do not inject drugs (non-PWID), PWID have a higher overdose rate [8] and are more likely to relapse [8]. Based on input from stakeholder PWID, we assumed that the working familiarity with injections gives PWID a higher probability of successful INF NLX kit administration during an overdose event compared to non-PWID. We estimated that 60% of PWID use Syringe Services Programs (SSP) by comparing SSP administrative records with the estimated number of PWID in CT. Because some SSP in Connecticut directly provide NLX kits, we assumed PWID using SSP were more likely to receive NLX kits than PWIDs not using SSP or non-PWID with OUD.

The model was calibrated by comparing overdose deaths from 2012 to 2020 for observed versus expected data (see Additional file 1: Appendix for details). We conducted simulations for 100,000 individuals to reduce random variations before scaling the values back to the study cohort size for overdose deaths. The model was developed using Microsoft Visual Studio Community 2022. Code was written in C/C++. Computations were conducted on Big Purple, the High-Performance Computing Facility at NYU Langone Medical Center.

### Model rates, costs, and utilities

Model input parameters were derived from published literature, community organizations, and expert opinions (Table 1; Additional file 1: Appendix). Modeled types of MOUD and proportions in the community and incarceration included methadone (30%), oral buprenorphine (60%), injectable buprenorphine (5%), and injectable naltrexone (5%). During incarceration, 100% of males received methadone and  70% of females received methadone, and 30% of all individuals received buprenorphine, based on estimates from CT DOC. Transition rates between the different treatment and opioid use statuses in the community were estimated from published literature and adjusted so that the modeled MOUD coverage level matched community MOUD coverage levels, while also satisfying expert opinion-informed criteria that: (1) approximately 20% of community-dwelling people under age 50 with OUD are −MOUD +remission, and (2) a decline in opioid use after age 50 converges to approximately 50% −MOUD +remission with increasing age [49].

To capture real-world heterogeneity among people with OUD, we divided the modeled population into higher-relapse propensity and lower-relapse propensity subgroups, with correspondingly differential transition rates to remission states. Based on the literature, transition rates from +MOUD states could vary by MOUD type (Additional file 1: Appendix). Finally, we modeled the tenfold higher relapse rate in the month following incarceration [25, 50–52].

Costs were derived from CT DPH and DOC partners, community organizations, and published literature, converted into 2021 US dollars and discounted at an annual rate of 3%. Healthcare costs include costs of MOUD and NLX, as well as services related to an overdose in the community (i.e., emergency medical services, emergency department admittance). Base case analyses were performed from a public payor perspective, including incarceration costs and health costs. The societal perspective costs additionally include crime costs for −MOUD−remission among people with OUD in the community and productivity loss costs for people with OUD when incarcerated and −MOUD-remission in the community. We assumed that individuals in the community who are treated with MOUD or −MOUD +remission do not incur crime costs or productivity loss costs.

Utilities were derived from published literature and substance use expert opinions. We assumed a 0.82 baseline utility for people treated with MOUD or who are −MOUD +remission based on Rhee and Rosenheck 2019 (Table 1) [40].

### Description of strategies

We compared maximum scale-up of three overdose prevention strategies: (a) distributing NLX in the community); (b) providing MOUD in the community (MOUD-COM); and (c) providing MOUD during incarceration (MOUD-INC) in the CT DOC. We assessed each of the three strategies independently or in combination. We compared scenarios of *maximizing* versus *current levels*. *Current levels* reflect practice at the time of writing in CT and are specified as follows: approximately 40% of POUD in the community use MOUD, and 10–40% (highest for SSP distribution to PWID) receive a NLX kit annually. Probability of receiving MOUD after incarceration is dependent on MOUD/remission status prior to incarceration: 1% if previously in the −MOUD +remission, 30% if previously in −MOUD +remission, and 43% if previously in +MOUD ± remission.

The MOUD strategies consist of specifiable proportions of methadone, buprenorphine-naloxone, extended-release naltrexone, and extended-release buprenorphine, at an assumed constant ratio during maximization. The NLX distribution strategy consists of varying proportions of INF and IN forms. The ratio between these proportions was assumed to remain constant during maximization. Provision of NLX occurs in the model both at release from incarceration and in the community. However, distribution of NLX at release was assumed to be conditional on receiving MOUD during incarceration, and hence was not modeled as an independent intervention strategy.

### Modeled scenarios

“Maximizing MOUD-INC” results in all incarcerated people with OUD receiving MOUD regardless of their MOUD status prior to incarceration. “Maximizing MOUD-COM” increases MOUD coverage to reach nearly all community dwelling people with OUD who are not in remission (80%). Because people in CT often do not have access to the MOUD type of their choice, re-assignment of MOUD type was possible every time an individual initiated or reinitiated MOUD in the community, upon incarceration, and upon release. “Maximizing NLX” results in nearly 100% of community-dwelling people with OUD receiving NLX kits at least annually.

### Sensitivity analyses

We conducted both one-way and probabilistic sensitivity analyses. In one-way sensitivity analysis, we tested how variations in some key inputs affect the number of overdose deaths averted by maximizing NLX and MOUD-COM. In probabilistic sensitivity analysis, values were randomly drawn from a distribution around each of the inputs for 10,000 simulations. Distributions were beta distribution for proportions and probabilities; lognormal for transition rates, costs, and utility decrements; and normal for utility and rate ratios. The results of these simulations were used to generate cost-effectiveness acceptability curves (CEACs) demonstrating the probability of cost-effectiveness of each intervention strategy at different willingness-to-pay thresholds.

## Results

Without any change in overdose prevention strategies, the simulated cohort will live an additional 32.7 years and accrue 26.1 QALYs (Table 2). An estimated 4711 overdose deaths would occur over 5 years and 11,655 overdose deaths are predicted during the stimulated cohort’s lifetime.

**Table 2:** Base case results (per CT people with OUD population)

Outcome (from simulation start)		
	Mean	Median
LY*	32.7	33.9
Discounted LY	18.9	21.4
QALYs	26.1	27.0
Discounted QALYs	15.0	17.0
Reincarcerations per person	3	0

Overdose deaths		
	5 year	Lifetime
Number	4711	11,655

Costs per Person (USD, 2021)		
	5 year	Lifetime
Health care perspective	$15,000	$77,000
Health care perspective, discounted	$14,000	$48,000
Societal perspective	$164,000	$829,000
Societal perspective, discounted	$154,000	$511,000

*Life expectancy from birth = 73.4

**Fig. 2:**
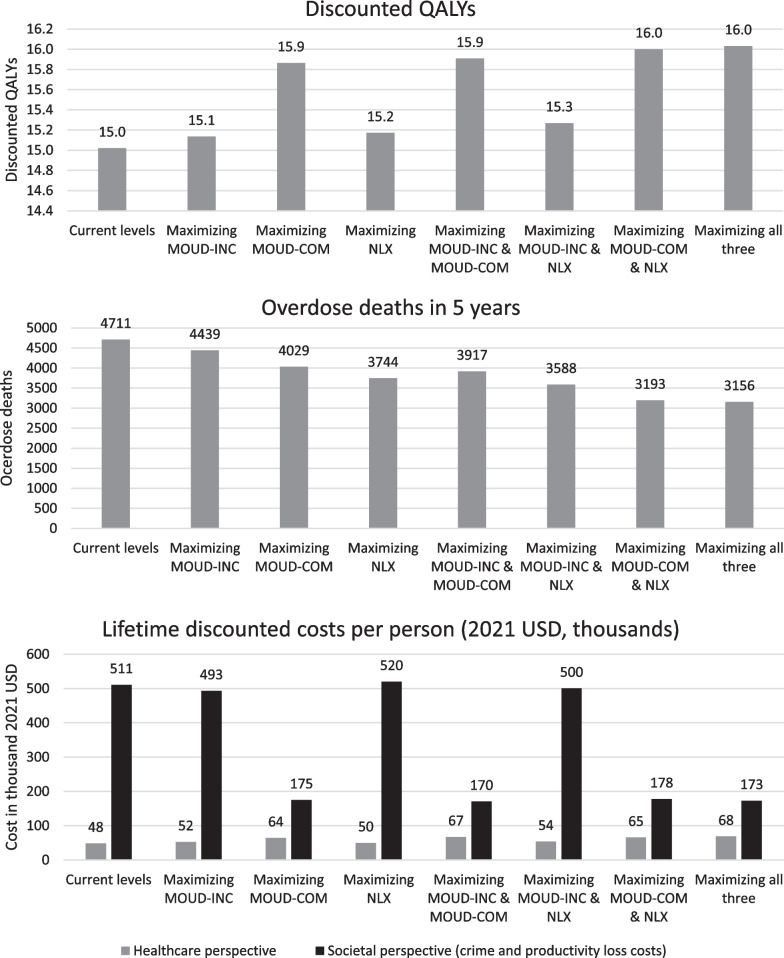
Results

**Fig. 3:**
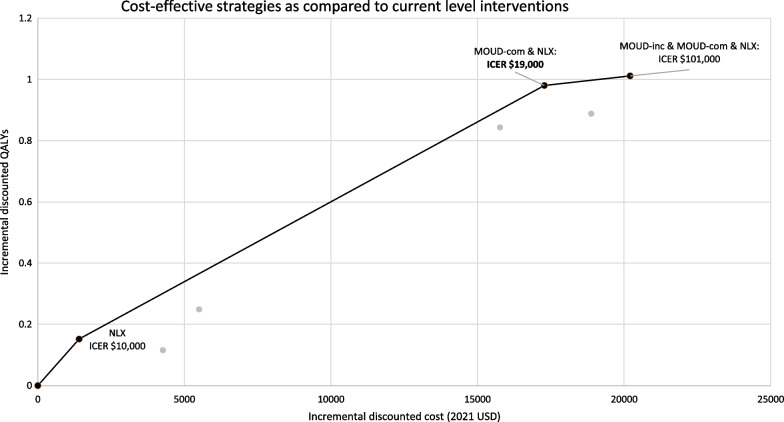
Strategies on the efficient frontier, as compared to current level interventions. Legend: (1) MOUD-COM: MOUD in community; MOUD-INC: MOUD in incarceration; NLX: naloxone in community (2) Maximized interventions are noted

Maximally scaling individual overdose prevention approaches could increase life expectancy and quality of life while also reducing overdose mortality. Maximizing NLX distribution reduces overdose deaths by 20% (967 deaths averted, Fig. 2), adding 0.4 LYs and 0.3 QALYs with a favorable ICER of $9000 per QALY (Fig. 3). By distributing INF rather than IN naloxone, an estimated 240 additional overdose deaths (25% reduction) could be prevented over 5 years, which would decrease medication costs without increasing overall costs. Maximizing MOUD-COM reduces overdose deaths by 14% (682 deaths averted), adding 1.5 LYs and 1.8 QALYS with a favorable ICER of $19,000 per QALY. Maximizing MOUD-INC reduces overdose deaths by 6% (272 deaths averted) over 5 years, adding 0.2 LYs and 0.2 QALYs with a favorable ICER of $37,000 per QALY.

Maximally scaling multiple interventions further increased benefits. Of all permutations, the most beneficial option that remained cost effective was to jointly maximize NLX and MOUD-COM, which reduced overdose deaths by 32% (1518 deaths averted) and added 1.8 LYs and 2.0 QALYs at a favorable ICER of $19,000 per QALY (Fig. 3). Additionally maximizing MOUD-INC modestly increased benefit but with a borderline ICER of $94,000 per QALY.

In sensitivity analyses, applying a societal perspective rather than a health sector perspective had a transformative impact on results, with all maximal scale-up scenarios becoming cost saving. Maximally scaling all interventions simultaneously saved society $338,000 per capita while reducing 5-year overdose deaths by 33% (Fig. 2c, calculated as the difference between “Current levels” and “Maximizing all three”).

**Fig. 4:**
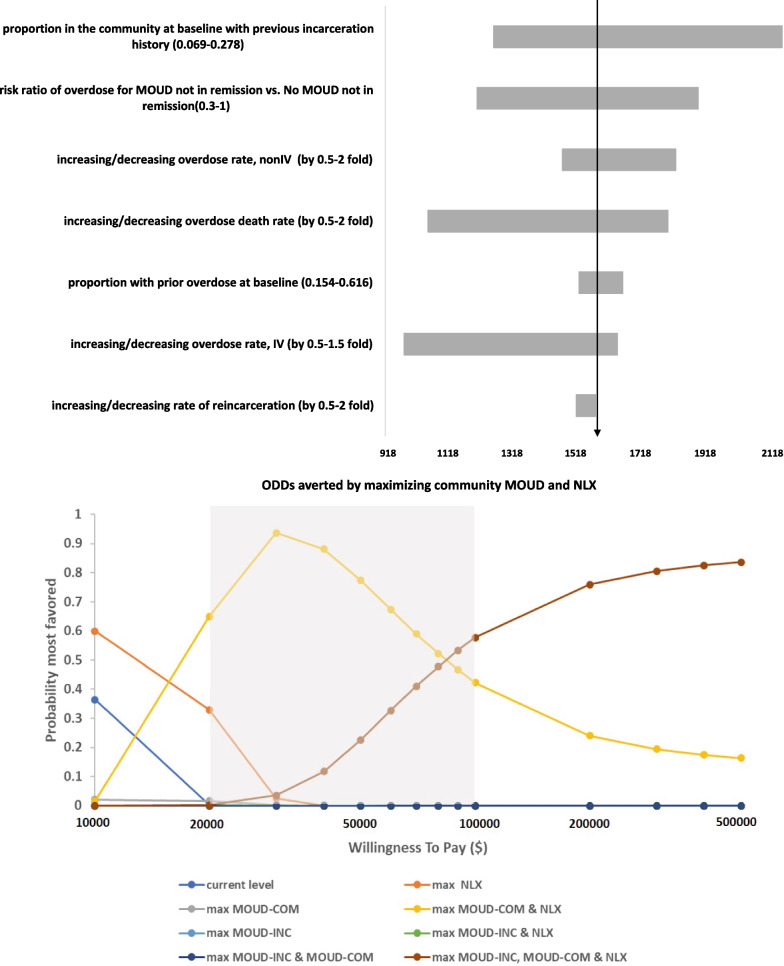
**a** Deterministic sensitivity analysis, overdose deaths averted in 5 years. **b** Cost-effectiveness acceptability curve

In one-way sensitivity analyses, estimates for overdose deaths averted were generally robust, including to uncertainty surrounding the rates of overdose among PWID and reincarceration, the proportion who experienced a prior overdose, or had been previously incarcerated (Fig. 4a). The uncertainty that had the greatest impact on overdose death projections was the ratio of overdose between those on MOUD and using opioids and those not treated with MOUD and using opioids, as well as the rate of fatal overdose overall and among people who do not inject. Probabilistic sensitivity analyses (Fig. 4b) indicated that maximizing MOUD-COM and NLX was cost effective with high certainty, even when varying all inputs simultaneously across their plausible ranges and even when exploring a wide range of willingness to pay for health benefits.

## Discussion

The results of this simulation study suggest that one-third of overdose deaths could be prevented within 5 years and 2 years of life expectancy could be gained by maximizing distribution of naloxone and MOUD capacity in the community. This approach was cost effective from a health sector perspective and cost saving from a societal perspective because of increased productivity and reduced crime. Our results indicate that substantial impact on the opioid overdose crisis can be achieved cost effectively in Connecticut and potentially throughout the United States by maximizing access to existing evidence-based interventions.

Our findings demonstrate that Connecticut’s efforts to increase the distribution of naloxone, including to people at elevated risk of an overdose, have already made strides in reducing overdose mortality and that additional efforts could build upon this success. We estimated that approximately 20% of overdose deaths could be prevented by maximizing naloxone distribution alone compared to its current levels, and most of those averted overdose deaths are attributed to targeting distribution to PWID. These results support that optimal naloxone distribution includes targeting the individuals who are at high risk of overdose [8, 10]. For example, an agent-based modeling study found that distributing naloxone in the community through pharmacies combined with distribution through SSP could reduce overdose deaths by 65% relative to no naloxone distribution [7]. However, a recent study that modeled the types of opioid epidemics (e.g., fentanyl, heroin, prescription opioids) and naloxone access in twelve representative US states found that only one state had sufficient naloxone access to achieve targeted levels of availability during witnessed overdoses [53]. Future comparative effectiveness and implementation science research is needed to determine the best strategies for community naloxone distribution, including where and to whom.

Moreover, individually maximizing naloxone led to the largest reduction in overdose mortality in the population compared to individually maximizing MOUD either in the community or carceral settings. This may run counter to what one may hypothesize the effects would be based on studies conducted at the individual level. A recent meta-analysis of clinical trials and observational studies assessing the impact of MOUD on all-cause and cause-specific mortality found that MOUD reduced drug-related deaths by almost 60% [54], whereas a recent systematic review reported that naloxone reduces overdose mortality by 30–50% depending on the availability of opioid education and naloxone distribution [55]. Yet our results are primarily aligned with those from other simulation studies modeling the population level effects, which have shown a substantial reduction in overdose mortality following expansion of naloxone and that those reductions are often greater than those achieved through other strategies such as increased MOUD [27, 56–58]. For example, a recent study that modeled the effectiveness of various opioid overdose interventions found that expanding naloxone availability would have the largest impact on mortality. Specifically, expanding naloxone by 30% would reduce overdose deaths 26% in the next 5 years while other interventions would have positive but smaller effects, such as a 25% increase in MOUD initiation leading to a 2% reduction in overdose [58]. However, other modeling studies have found that treatment expansion reduces a greater proportion of overdose deaths compared to naloxone [59]. Additional experimental, observational, and simulation modeling research is clearly needed to determine the most effective interventions for both the individual and the population, including research that elucidates the mechanisms of effect at both levels.

As has also been demonstrated in other simulation modeling studies [27, 59], we found that combining the prevention strategies led to the greatest effects on the outcomes. We found that maximally scaling both community naloxone distribution and treatment with MOUD maximized the benefits gained while maintaining cost effectiveness. This multi-pronged approach may be optimal because it combines tertiary prevention of reversing a potentially fatal overdose with secondary prevention of treating OUD to reduce overdose risk. People with OUD treated with MOUD are less likely to overdose [60, 61] but unfortunately, most people with OUD do not receive MOUD. In the US, up to 87% of people with OUD who may benefit from MOUD do not receive it [32]. In Connecticut, this statistic is 54–68%, which is better than national averages but still offering potential for improvement. The reasons for the lack of treatment with MOUD are wide-ranging, including financial barriers, scarcity of providers [62], and lack of perceived need for treatment [63]. Together with expanding community MOUD coverage, reframing MOUD as a form of harm reduction itself that can support non-abstinent-based goals may help to increase uptake to reduce overdose mortality [64].

The findings from our study support other reports that achieving the goal of 40% reduction in opioid overdose by 2025 [65] will require an array of effective OUD and overdose prevention strategies across sectors. Our finding of a 33% reduction in 5-year overdose death reduction is comparable to the predicted 40% reduction in overdose deaths in Massachusetts, which was achieved by combining maximal scaling of naloxone distribution, MOUD initiation, and treatment retention [28]. In a simulation model that tested the effects of 11 high-impact overdose prevention strategies on reducing OUD prevalence and overdose mortality within 10 years, results suggested that a multifaceted approach featuring interventions that specifically focused on reducing fentanyl-related increased overdose risk, increasing naloxone distribution, and increasing support for people in recovery saved the most lives [27]. These findings suggest that maximizing availability of existing primary, secondary, and tertiary overdose prevention approaches will save lives and money, and continued efforts are needed to address the barriers to their implementation.

The US opioid crisis is constantly and rapidly evolving, and an important limitation of our study is that some of the literature used for model inputs was from research conducted when higher potency synthetic opioids and co-use of xylazine were less prevalent. For example, opioid overdose mortality in Connecticut rose over 10% between 2017 and 2021, likely driven by increasing fentanyl exposure. Moreover, we estimated that PWID have higher overdose rates compared to those who administer opioids through other routes, but recent evidence suggests that beginning in 2022, smoking overtook injection as the route of administration that accounted for the greatest proportion of overdose deaths, especially in deaths in which illicitly manufactured fentanyl was detected [66]. Additionally, we modeled that that opioid use decreased as individuals “age out” of substance use after peaks during young adulthood [67]. As the baby boomer generation enters older adulthood, prior trends in substance use and aging may no longer hold true and problematic opioid use and overdose have been increasing among older adults [68, 69]. The veterinary tranquilizer xylazine is increasingly detected in fatal heroin and fentanyl overdoses and Connecticut had among the highest rates of xylazine-involved deaths in 2022 [70], which is not explicitly captured in our model. However, our deterministic sensitivity analyses varied the model inputs, including rates of incarceration and overdose mortality, and suggests that our results are overall robust. Also, some of our parameter estimates, such as the effectiveness of naloxone, which may be reduced in the presence of fentanyl, are conservative compared to more recent evidence. Taken together, the dynamic nature of the US opioid crisis underscores that we must consider our findings in that context and highlights the crucial need for future research that is responsive to emerging issues in drug-related overdose prevention.

Further limitations are that assumptions made in favor of model parsimony do not reflect the complex reality of opioid use. For example, the model did not account for overdoses occurring in carceral settings which are commonly reported in the lay press but have not been identified in empirical research, thus limiting our ability to estimate the magnitude of the bias that this assumption may introduce. Moreover, in the model, one’s initial risk of return to opioid use (i.e., relapse propensity) did not vary although many factors, including treatment with MOUD, influence the potential for relapse over time [71, 72]. Finally, results are obtained based on simulations from a modest cohort in a specific geographic area and may not be generalizable.

In conclusion, a significant number of overdose deaths can be prevented among people with opioid use disorder in Connecticut by maximizing the availability of existing prevention strategies of community based MOUD and naloxone. This approach is cost effective from the healthcare perspective and cost saving from a societal perspective.

### Supplementary Information

**Additional file 1**. Technical Appendix.

## Data Availability

All inputs for the mathematical simulation model were derived from existing, secondary data, including published scientific articles, and publicly available state-level data for Connecticut. Modeling code used to generate modeling simulation outcomes is available upon request.

## Abbreviations

NLX: Naloxone
OUD: Opioid use disorder
MOUD: Medications for opioid use disorder
MOUD-INC: Medications for opioid use disorder for people who are incarcerated
MOUD-COM: Medications for opioid use disorder for people in the community
LY: Life-year
QALY: Quality-adjusted life year
US: United States
USD: United States dollars
PWID: People who inject drugs
IM: Intramuscular
IN: Intranasal
CT: Connecticut
DOC: Department of Corrections
DPH: Department of Public Health
SSP: Syringe service program
CEAC: Cost-effectiveness acceptability curve
EMS: Emergency Medical Services
