# Supplementary material for: Reducing overdose deaths among persons with opioid use disorder in connecticut
Source: Harm Reduct J. 2024 May 28;21:103. doi: 10.1186/s12954-024-01026-6 (PMC11131266; doi:10.1186/s12954-024-01026-6)
Supplement: Supplementary file 1 — Additional file 1. Technical Appendix. [file 12954_2024_1026_MOESM1_ESM.docx]

**Technical Appendix**

**Model methods and parameters**

Contents

[A1: Introduction 1](#_Toc494267838)

[A2: Characteristics of each hypothetical individual 1](#_Toc494267839)

[A3: The simulation procedure 2](#_Toc494267841)

[A4: Special treatments 5](#_Toc494267842)

[A5: Model Calibration 7](#_Toc494267843)

[A6: Sensitivity analysis 7](#_Toc494267844)

[A7: Development and computational environment 7](#_Toc494267845)

[References 8](#_Toc494267846)

[Figures 10](#_Toc494267847)

[Tables 12](#_Toc494267848)

# A1: Introduction

Our Monte Carlo model simulates a cohort of hypothetical individuals with opioid use disorder (OUD). Each individual is generated with an age at initiation, gender, opioid use status, and incarceration status. He/she then goes through a virtual life in the model, varying his/her conditions over time, until he/she dies.

# A2: Characteristics of each hypothetical individual

**Age**

All individuals in the model start with an age 20 or older.

**Gender**

Male or female.

**Opioid use status**

People with OUD (POUD) are divided into two groups: one with higher-relapse propensity (GROUP_HIGH_RELAP), the other with lower-relapse propensity (GROUP_LOW_RELAP). For GROUP_LOW_RELAP, people can transition between any of these 3 states: *on treatment and not using opioids* (+MOUD+remission), actively *using opioids without treatment* (-MOUD-remission), or *not using opioids without treatment* (-MOUD+remission). For GROUP_HIGH_RELAP, people can only transition between 2 states: *using opioids without treatment* (-MOUD-remission), and *using opioids with treatment* (+MOUD-remission).

In addition, POUD are divided into two types: PWID or non-PWID. Each type has different overdose rate, and rate of getting naloxone kit in the community.

**Incarceration status**

POUD may or may not be incarcerated at initiation. Incarcerations may recur. POUD are divided into two groups: one with higher reincarceration rate (GROUP_HIGH_REINC) and the other with lower rate (GROUP_LOW_REINC).

When POUD are incarcerated, the simulation assumes they are not actively using opioids. Rather, they are in forced remission, either with or without MOUD.

# A3: The simulation procedure

**Overview**

In each iteration of the simulation one hypothetical individual is generated, with a starting age, sex, initial settings of opioid use status and incarceration status. As shown in figure A1, once the status of this person is initialized, he/she enters into a virtual life composed of many cycles. The cycle length is set to be a day. In each day this person goes through the following steps:

1. Increase the age by one day.
2. Update incarceration status: if incarcerated, is it time to be released? If not incarcerated, is this person incarcerated in this cycle?
3. Update opioid use condition: if newly incarcerated, this person will be assigned to either +MOUD+remission or -MOUD+remission and he/she will stay in that status while incarcerated. POUD in community can transition between different states.
4. Check if an overdose event happens when this person is actively using opioid (whether on treatment). This person may or may not die of overdose depending on whether naloxone is used, EMS is called, etc. If an overdose death happens, we terminate this iteration and move on to a new individual.
5. If this person does not die of overdose, check if he/she dies of any other reasons (baseline mortality). If this person dies, we terminate this iteration and move on to a new individual.
6. If this person does not die of any cause in this cycle, move on to the next cycle (go back to step 1).

After a desired number of individuals have been simulated (for this study, we used 100,000), we can perform various statistical analyses of the cohort. Details of each step are described below.

**Initialization of incarceration/opioid use status**

When a new individual is generated, the first characteristic to define is age at initiation. In this study, we simulated a cohort with a specific age distribution. There are 5 age groups: 20-29, 30-39, 40-49, 50-59, and 60+, with the following proportions for each group: 0.118, 0.412, 0.262, 0.172, 0.036.(1) First the age group is randomly assigned, then a specific age is drawn using a uniform distribution for the age range of that age group.

The second characteristic is incarceration status. For the current study, 3% starts incarcerated and 33.5% (CTDOC) of them are randomly chosen to go to jail while the rest go to prison. Then based on the current MOUD coverage in jail/prison, 26.4% (CTDOC) of them are randomly assigned as +MOUD+remission while the rest are -MOUD+remission. For those starting in community, 20% are in state -MOUD+remission (expert opinion), 16.8% (expert opinion; DMHAS) on MOUD, and the rest are -MOUD-remission. Additionally, 13.9% (2) of those starting in community are randomly chosen to have previous incarceration history.

The third characteristic is whether this person is a PWID. Among those who are incarcerated at initialization and those who are in the community but with precious incarceration history, 30% (expert opinion) are randomly assigned to be a PWID. Among those in the community without any incarceration record, 5% (expert opinion) are randomly chosen to be a PWID.

Finally, this person is randomly assigned to GROUP_LOW_RELAP or GROUP_HIGH_RELAP depending on whether he/she is PWID. 20% of PWID and 60% of non-PWID belong to GROUP_LOW_RELAP (expert opinion).

**Release and reincarceration**

If the person is incarcerated, the model checks the duration of the incarceration and if it is equal to the defined length of incarceration (Jail: 25 days, Prison: 949 days, from CTDOC), this person will be released.

If the person is not incarcerated, the model checks if he/she will be reincarcerated. Annual rate of being reincarcerated depends on OUD status, age, whether this person belongs to GROUP_HIGH_REINC or GROUP_LOW_REINC (Table A1). This rate is converted to a daily probability and a random number is generated to decide whether this person will be reincarcerated.

**Change of OUD status from community to incarceration**

When POUD in community get incarcerated, they have to choose and stay in one of the two states: +MOUD+remission or -MOUD+remission. According to a person’s OUD status in the community, the probability of choosing MOUD varies. Based on the current CTDOC MOUD coverage, the probabilities are: for those -MOUD+remission in the community: 1%; for those -MOUD-remission in the community: 30%; for those +MOUD+/-remission: 43%.

**Change of OUD status from incarceration to community**

When incarcerated POUD are released, if they are of GROUP_LOW_RELAP, they will be probabilistically put into any of the three states: +MOUD+remission, -MOUD+remission, and -MOUD-remission. For those of GROUP_HIGH_RELAP, they will be probabilistically put into one of the two states: +MOUD-remission, and -MOUD-remission. All probabilities are listed in Table A2. Note that the probabilities vary by MOUD type if people are on MOUD while incarcerated.

**Change of OUD status in community**

POUD in community who are in GROUP_LOW_RELAP can transition between any of the three states: +MOUD+remission, -MOUD+remission, and -MOUD-remission. POUD in the community who are in GROUP_HIGH_RELAP can only transition between two states: +MOUD-remission, and -MOUD-remission. All transitions are controlled by daily probabilities derived by annual rates, which are defined in Table A3. Note that within the first month post release, the rates of relapse from MOUD are 10 times higher than afterwards. For transitions from an on MOUD status, the rates vary by MOUD type.

**Check overdose events and overdose deaths**

The simulation assumes that overdose events are not possible when a person is incarcerated. POUD in community are at risk of overdose if they are -MOUD-remission, with overdose rates varying by whether an individual is PWID and whether he/she has prior overdose (Table A4). In addition, those of GROUP_HIGH_RELAP and +MOUD-remission can also overdose, but with a 38% (3)reduction in overdose rates compared to those who are -MOUD-remission.

If an overdose event happens, it might be witnessed, with a probability of 0.79.(4) For overdose events witnessed, if naloxone kit is available, there is a possibility that the kit is successfully used (probabilities vary by kit type and OUD type, Table A4). Also, if an overdose is witnessed, EMS might be called with a probability of 0.6.(5-7) The probability that an overdose is fatal depends on whether a naloxone kit is successfully used and whether EMS is called (Table A4).

**Check mortality of all other causes**

If a person does not die of overdose, the model checks if he/she dies of any other causes. Mortality rates are calculated using weighted average of rates from three race/ethnic groups based on their representation in the target population: White (59.2%), Black (10.5%), and Hispanic (30.3%).(1) We used 2017 mortality from the National Vital Statistics report.(8) We also added a non-overdose excess mortality among people with OUD: 0.00978 if -MOUD-remission and 0.00318 if taking MOUD or in remission.(9)

# A4: Special treatments

**Aging out**

POUD of GROUP_LOW_RELAP may “age out” of opioid use (expert opinion). Therefore, once people in this group turn age 50, their transition rates from -MOUD+remission to both +MOUD+remission and -MOUD-remission are set to 0.

**MOUD type**

Four types of MOUD are simulated in the model: methadone (METH), buprenorphine (BUP), naltrexone (NAL) and injectable buprenorphine (INJBUP). When a person starts on MOUD, he/she will be randomly assigned to one type of MOUD depending on proportions for each type (Table A5).

When a person is of GROUP_HIGH_RELAP, being on MOUD is not in remission and therefore overdose can still happen. However, MOUD reduces the probability of an overdose event. In the model, each form of MOUD reduces overdose risk by 38%, with one exception: for the first 4 weeks after starting METH, rate of overdose is the same as those not on MOUD. The differential impact of MOUD on overdose is included in the model through the retention rates of each type of MOUD.

**Naloxone distribution**

Naloxone distribution can happen at jail/prison release or in community. At jail/prison release, anyone who is on MOUD while incarcerated is given a naloxone kit. In community, PWID in Syringe Service Programs (SSP) have a higher probability of getting a naloxone kit (annual rate of 0.51) than PWID not in SSP and non_PWID (0.1), based on expert opinion. Each day, a POUD in community who does not own a kit will have a probability of getting a kit. Once a kit is given, it will stay with the owner until one of the following two things happen: 1) this person is incarcerated; 2) this person has an overdose event that is witnessed and therefore the kit is assumed to be used.

Two types of naloxone are available: intranasal (IN_NLX) and intramuscular (IM_NLX). At release from incarceration, only IN_NLX are given. In community, only IN_NLX are distributed in this study, but the simulation allows for the possibility that IM_NLX can be given to PWID.

**Calculation of costs**

Cost is accumulated each day according to the status of the patient. If a person is incarcerated, there are incarceration costs, productivity loss costs and cost of MOUD (if this person is on MOUD). If a person is in the community and -MOUD-remission, there are crime costs and productivity loss costs. If a person is in the community and on MOUD, cost of MOUD is counted.

Each time before a person starts on BUP, a liver function test is required. Therefore, the cost of this test ($11 per test;(10) is counted.

Whenever a naloxone kit is distributed, the cost of the kit is counted. If an overdose event happens, EMS cost and ED admission cost may be incurred depending on whether EMS is called and/or this person is sent to ED.

Costs for MOUD are listed in Table A6. The other costs are listed in the main manuscript Table 1.

**Scaling up policy options**

Maximizing MOUD-INC results in the probability of choosing MOUD when POUD get incarcerated being set at 100% regardless of their OUD status in the community.

Maximizing MOUD-COM results in 1) the transition rate from +MOUD+/-remission to -MOUD-remission being set to 0; 2) the transition rate from -MOUD+remission to -MOUD-remission also being set to 0; 3) the transition rate from +MOUD+remission to -MOUD+remission being doubled; 4) the transition rate from -MOUD+remission to +MOUD+remission being increased by 1.25 fold; 5) for those who are -MOUD+remission while incarcerated, the probability of going to -MOUD-remission after release being set to 0. These adjustments were empirically back-calculated to asymptotically put on treatment every POUD not in remission.

Maximizing NLX-COM results in the rate of getting NLX for PWID in SSP increasing by multipliers of 200-fold, and for PWID not in SSP and non-PWID, increasing by multipliers of 1000-fold, adjustments empirically back-calculated to asymptotically give every POUD in the community a NLX kit.

# A5: Model Calibration

We collected historical data for number of overdose deaths, community MOUD coverage, community naloxone kit coverage for most of the years from 2012-2020. For each year, we adjusted the transition rate from -MOUD-remission to +MOUD+/-remission to reflect the community MOUD coverage of that year, and likewise the rate of getting a kit in community for community naloxone coverage. Then we tried to produce a number of overdose deaths in the first year that matches the number of ODD reported for that year by adjusting only one parameter: the probability of overdose for PWID, because the fentanyl mortality burden is disproportionately concentrated among PWID. Comparison of number of ODD between data and model is shown in Figure A2.

# A6: Sensitivity analysis

We conducted both one-way and probabilistic sensitivity analysis. In one-way sensitivity analysis, we tested how variations in some key inputs affect the number of overdose deaths averted by maximizing MOUD-COM and NLX. For each variable, we ran two analyses (one at current level MOUD-COM and NLX, the other with maximized MOUD-COM and NLX) twice (the first time using a lower bound value, and the second time an upper bound value of the variable while holding all other variables unchanged).

In probabilistic sensitivity analysis, values were randomly drawn from a distribution around each of the inputs (those listed with ranges in the main manuscript Table 1) for 10,000 times. Distributions were beta distribution for proportions, probabilities, utilities, and utility decrements, lognormal for transition rates, and costs, and normal for rate ratios. For each set of randomly drawn inputs, 8 analyses were run to test all permutations of scaling up policy options. Cost-effectiveness frontiers were then calculated for all 10,000 sets of parameters in order to generate the cost-effectiveness acceptability curves (CEACs).

# A7: Development and computational environment

The model was developed using Microsoft Visual Studio Community 2022. Code was written in C/C++. Computations were conducted on Big Purple, the High-Performance Computing Facility at NYU Langone Medical Center. The calculation of frontiers and CEACs were conducted using MatLab.

# References

1. Haas A, Viera A, Doernberg M, Barbour R, Tong G, Grau LE, et al. Post-incarceration outcomes for individuals who continued methadone treatment while in Connecticut jails, 2014-2018. Drug Alcohol Depend. 2021;227:108937.

2. Winkelman TNA, Chang VW, Binswanger IA. Health, Polysubstance Use, and Criminal Justice Involvement Among Adults With Varying Levels of Opioid Use. JAMA Netw Open. 2018;1(3):e180558.

3. Larochelle MR, Bernson D, Land T, Stopka TJ, Wang N, Xuan Z, et al. Medication for Opioid Use Disorder After Nonfatal Opioid Overdose and Association With Mortality. Annals of Internal Medicine. 2018;169(3):137-45.

4. Townsend T, Blostein F, Doan T, Madson-Olson S, Galecki P, Hutton DW. Cost-effectiveness analysis of alternative naloxone distribution strategies: First responder and lay distribution in the United States. Int J Drug Policy. 2020;75:102536.

5. Galea S, Worthington N, Piper TM, Nandi VV, Curtis M, Rosenthal DM. Provision of naloxone to injection drug users as an overdose prevention strategy: early evidence from a pilot study in New York City. Addict Behav. 2006;31(5):907-12.

6. Bennett T, Holloway K. The impact of take-home naloxone distribution and training on opiate overdose knowledge and response: An evaluation of the THN Project in Wales. Drugs: Education, Prevention and Policy. 2012;19(4):320-8.

7. Lankenau SE, Wagner KD, Silva K, Kecojevic A, Iverson E, McNeely M, et al. Injection drug users trained by overdose prevention programs: responses to witnessed overdoses. J Community Health. 2013;38(1):133-41.

8. Arias, E, Xu, J. United States Life Tables, 2017. Centers for Disease Control and Prevention, Statistics DoV; 2019. Contract No.: 7.

9. Fairley M, Humphreys K, Joyce VR, Bounthavong M, Trafton J, Combs A, et al. Cost-effectiveness of Treatments for Opioid Use Disorder. JAMA Psychiatry. 2021;78(7):767-77.

10. College of American Pathologists. Medical Clinical Laboratory Fee Schedule. 2017.

11. National Center for Health Statistics. VSSR Provisional Drug Overdose Counts. Available from https://data.cdc.ov/d/xkb8-kh2a

12. Westerberg VS, McCrady BS, Owens M, Guerin P. Community-Based Methadone Maintenance in a Large Detention Center is Associated with Decreases in Inmate Recidivism. J Subst Abuse Treat. 2016;70:1-6.

13. Hunt KE, B. The Effects of Aging on Recidivism Among Federal Offenders. United States Sentencing Commission; 2017.

14. Durose MR CA, Snyder HN. Recidivism of Prisoners Released in 30 States in 2005: Patterns from 2005 to 2010. U.S. Department of Justice, Statistics BoJ; 2014.

15. Gordon MS, Kinlock TW, Schwartz RP, Fitzgerald TT, O'Grady KE, Vocci FJ. A randomized controlled trial of prison-initiated buprenorphine: prison outcomes and community treatment entry. Drug Alcohol Depend. 2014;142:33-40.

16. Lee JD, McDonald R, Grossman E, McNeely J, Laska E, Rotrosen J, et al. Opioid treatment at release from jail using extended-release naltrexone: a pilot proof-of-concept randomized effectiveness trial. Addiction. 2015;110(6):1008-14.

17. Clark CB, Hendricks PS, Lane PS, Trent L, Cropsey KL. Methadone maintenance treatment may improve completion rates and delay opioid relapse for opioid dependent individuals under community corrections supervision. Addict Behav. 2014;39(12):1736-40.

18. Mattick RP, Breen C, Kimber J, Davoli M. Methadone maintenance therapy versus no opioid replacement therapy for opioid dependence. Cochrane Database Syst Rev. 2009;2009(3):CD002209.

19. Nielsen S, Larance B, Degenhardt L, Gowing L, Kehler C, Lintzeris N. Opioid agonist treatment for pharmaceutical opioid dependent people. Cochrane Database Syst Rev. 2016(5):CD011117.

20. Minozzi S, Amato L, Vecchi S, Davoli M, Kirchmayer U, Verster A. Oral naltrexone maintenance treatment for opioid dependence. Cochrane Database Syst Rev. 2011;2011(4):CD001333.

21. Lobmaier P, Kornor H, Kunoe N, Bjorndal A. Sustained-release naltrexone for opioid dependence. Cochrane Database Syst Rev. 2008(2):CD006140.

22. Comer SD, Sullivan MA, Yu E, Rothenberg JL, Kleber HD, Kampman K, et al. Injectable, sustained-release naltrexone for the treatment of opioid dependence: a randomized, placebo-controlled trial. Arch Gen Psychiatry. 2006;63(2):210-8.

23. Indivior I. A Randomized, Double-Blind, Placebo-Controlled, Multicenter Study To Assess the Efficacy, Safety, and Tolerability of Multiple Subcutaneous Injections of Depot Buprenorphine (RBP-6000 [100 mg and 300 mg]) Over 24 Weeks in Treatment-Seeking Subjects With Opioid Use Disorder. Clinical trial registration. clinicaltrials.gov; 2018 2018/02/16/. Report No.: NCT02357901.

24. Haight BR, Learned SM, Laffont CM, Fudala PJ, Zhao Y, Garofalo AS, et al. Efficacy and safety of a monthly buprenorphine depot injection for opioid use disorder: a multicentre, randomised, double-blind, placebo-controlled, phase 3 trial. Lancet. 2019;393(10173):778-90.

25. Schwartz RP, Kelly SM, O'Grady KE, Gandhi D, Jaffe JH. Randomized trial of standard methadone treatment compared to initiating methadone without counseling: 12-month findings. Addiction. 2012;107(5):943-52.

26. Hser YI, Hoffman V, Grella CE, Anglin MD. A 33-year follow-up of narcotics addicts. Arch Gen Psychiatry. 2001;58(5):503-8.

27. Ling W, Nadipelli VR, Aldridge AP, Ronquest NA, Solem CT, Chilcoat H, et al. Recovery From Opioid Use Disorder (OUD) After Monthly Long-acting Buprenorphine Treatment: 12-Month Longitudinal Outcomes From RECOVER, an Observational Study. J Addict Med. 2020;14(5):e233-e40.

28. Kilaru AS, Xiong A, Lowenstein M, Meisel ZF, Perrone J, Khatri U, et al. Incidence of Treatment for Opioid Use Disorder Following Nonfatal Overdose in Commercially Insured Patients. JAMA Netw Open. 2020;3(5):e205852.

29. Krieter P, Chiang N, Gyaw S, Skolnick P, Crystal R, Keegan F, et al. Pharmacokinetic Properties and Human Use Characteristics of an FDA-Approved Intranasal Naloxone Product for the Treatment of Opioid Overdose. J Clin Pharmacol. 2016;56(10):1243-53.

30. Dietze P, Jauncey M, Salmon A, Mohebbi M, Latimer J, van Beek I, et al. Effect of Intranasal vs Intramuscular Naloxone on Opioid Overdose: A Randomized Clinical Trial. JAMA Netw Open. 2019;2(11):e1914977.

31. Murphy SM, McCollister KE, Leff JA, Yang X, Jeng PJ, Lee JD, et al. Cost-Effectiveness of Buprenorphine-Naloxone Versus Extended-Release Naltrexone to Prevent Opioid Relapse. Ann Intern Med. 2019;170(2):90-8.

32. Clemans-Cope L, Winiski E, Epstein M, Basurto L. Medicaid prescriptions for extended-release medications to treat opioid use disorder: State trends from 2011 to 2018. The Urban Institute; 2020.

# Figures


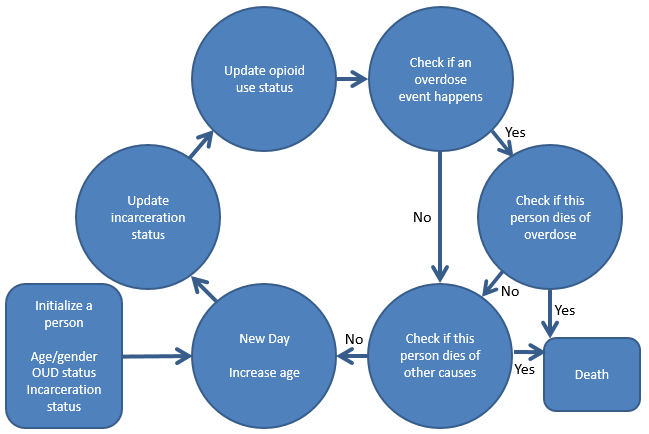


Figure A1: Algorithmic flow chart for a simulated person


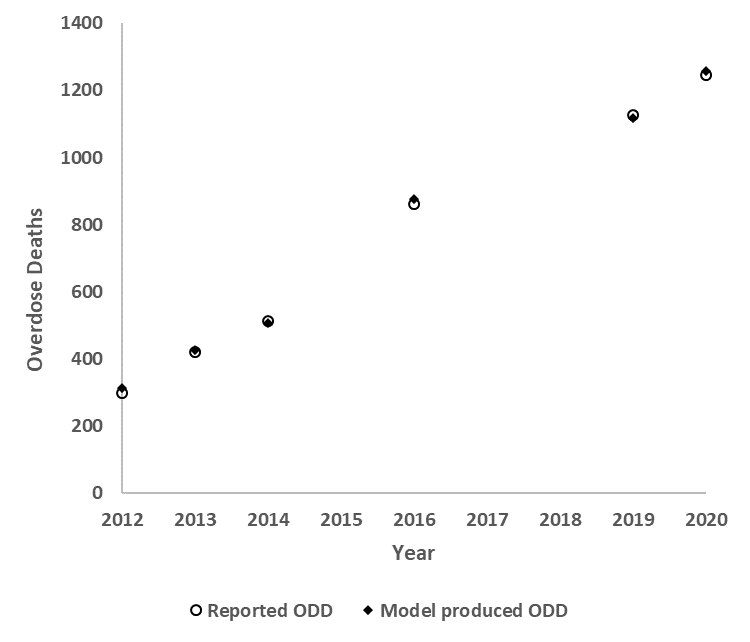


Figure A2: Calibration results: model produced ODD vs. reported ODD between 2012-2020. Reported ODD are from National Vital Statistics System provisional drug overdose death counts(11)

# Tables

Table A1: rate of reincarceration(12-14)

|  | active | | | on MOUD | | | abstinent | | |  |
| --- | --- | --- | --- | --- | --- | --- | --- | --- | --- | --- |
|  | | **GROUP_HIGH_REINC** | **GROUP_LOW_REINC** | | **GROUP_HIGH_REINC** | **GROUP_LOW_REINC** | | **GROUP_HIGH_REINC** | **GROUP_LOW_REINC** | |
| age<21 | | 4.084624 | 0.953079 | | 3.500729 | 0.816837 | | 3.294648 | 0.768751 | |
| 21-24 | | 4.511481 | 1.052679 | | 3.866567 | 0.902199 | | 3.638950 | 0.849088 | |
| 25-29 | | 3.801313 | 0.886973 | | 3.257917 | 0.760181 | | 3.066130 | 0.715430 | |
| 30-34 | | 3.000000 | 0.700000 | | 2.571151 | 0.599935 | | 2.419793 | 0.564618 | |
| 35-39 | | 2.562743 | 0.597973 | | 2.196400 | 0.512493 | | 2.067103 | 0.482324 | |
| 40-44 | | 2.417462 | 0.564074 | | 2.071887 | 0.483440 | | 1.949919 | 0.454981 | |
| 45-49 | | 1.768116 | 0.412560 | | 1.515365 | 0.353585 | | 1.426158 | 0.332770 | |
| 50-54 | | 1.266849 | 0.295598 | | 1.085754 | 0.253343 | | 1.021838 | 0.238429 | |
| 55-59 | | 0.872315 | 0.203540 | | 0.747618 | 0.174444 | | 0.703608 | 0.164175 | |
| 60-64 | | 0.852009 | 0.198802 | | 0.730215 | 0.170383 | | 0.687229 | 0.160353 | |
| age>=65 | | 0.387218 | 0.090351 | | 0.331865 | 0.077435 | | 0.312329 | 0.072877 | |

Table A2: probabilities for transitions from incarceration to community (calculations derived from expert opinion and stakeholders in addition to cited sources)

| Status in incarceration | Status in community | MOUD type while incarcerated | | | |
| --- | --- | --- | --- | --- | --- |
|  |  | METH | BUP | NAL | INJBUP |
| For GROUP_LOW_RELAP | | | | | |
| On MOUD in remission | Not on MOUD in remission | 0.166 | 0.213(15) | 0.201(16) | 0.201(16) |
|  | On MOUD in remission | 0.534 | 0.403 | 0.436 | 0.436 |
|  | Not on MOUD not in remission | 0.300 | 0.384(15) | 0.363(16) | 0.363(16) |
| Not on MOUD in remission | Not on MOUD in remission | 0.153 | 0.153 | 0.153 | 0.153 |
|  | On MOUD in remission | 0.310 | 0.310 | 0.310 | 0.310 |
|  | Not on MOUD not in remission | 0.537 | 0.537 | 0.537 | 0.537 |
| For GROUP_HIGH_RELAP | | | | | |
| On MOUD in remission | On MOUD not in remission | 0.534 | 0.403 | 0.436 | 0.436 |
|  | Not on MOUD not in remission | 0.466 | 0.597 | 0.564 | 0.564 |
| Not on MOUD in remission | On MOUD not in remission | 0.310 | 0.310 | 0.310 | 0.310 |
|  | Not on MOUD not in remission | 0.690 | 0.690 | 0.690 | 0.690 |

Table A3: Calculated annual rates of transitions between different OUD statuses in community(3, 17-28)

| Starting from on MOUD | | | | | | | | | | | | | | |  |  |
| --- | --- | --- | --- | --- | --- | --- | --- | --- | --- | --- | --- | --- | --- | --- | --- | --- |
| Beginning status | Ending status | Low risk | | | | | High risk (within month post release) | | | | | | | |  |  |
|  |  | METH | BUP | NAL | | INJBUP | | METH | | BUP | NAL | | INJBUP | | | |
| For GROUP_LOW_RELAP | | | | | | | | | | | | | | | |  |
| **On MOUD in remission** | **Not on MOUD not in remission** | 0.127 | 0.329 | 0.323 | | 0.329 | | 1.27 | | 3.29 | 3.23 | | 3.29 |  |  |  |
| **On MOUD in remission** | **Not on MOUD in remission** | 0.315 | 0.158 | 0.160 | | 0.158 | | 0.007 | | 8.08E-06 | 0.160 | | 0.158 |  |  |  |
| For GROUP_HIGH_RELAP | | | | | | | | | | | | | | | |  |
| **On MOUD not in remission** | **Not on MOUD not in remission** | 0.127 | 0.329 | 0.323 | | 0.329 | | 1.27 | | 3.29 | 3.23 | | 3.29 |  |  |  |
| Starting from not on MOUD | | | | | | | | | | | | | | |  |  |
| Beginning status | Ending status | Low risk | | | High risk (within month post release) | | | | | | |  |  |  |  |  |
| For GROUP_LOW_RELAP | | | | | | | | | | | |  |  |  |  |  |
| **Not on MOUD not in remission** | **On MOUD in remission** | 0.34 | | | 0.34 | | | |  |  |  |  |  |  |  |  |
| **Not on MOUD not in remission** | **Not on MOUD in remission** | 1.07 | | | 1.07 | | | |  |  |  |  |  |  |  |  |
| **Not on MOUD in remission** | **Not on MOUD not in remission** | 0.01 | | | 0.1 | | | |  |  |  |  |  |  |  |  |
| **Not on MOUD in remission** | **On MOUD in remission** | 0.29 | | | 0.06 | | | |  |  |  |  |  |  |  |  |
| For GROUP_HIGH_RELAP | | | | | | | | | | | |  |  |  |  |  |
| **Not on MOUD not in remission** | **On MOUD not in remission** | 0.34 | | | 0.34 | | | |  | | |  |  |  |  |  |

Table A4. Inputs related to overdose (5-7, 29, 30)

| OUD type | Prior overdose | Annual rate of overdose | OUD type | Naloxone kit type | Probability kit successfully used | Kit successfully used | EMS called | Probability overdose being fatal |
| --- | --- | --- | --- | --- | --- | --- | --- | --- |
| PWID | N | 1.8 | PWID | Intranasal | 0.697 | N | N | 0.101 |
| PWID | Y | 5.6 | PWID | Intramuscular | 0.828 | Y | N | 0.021 |
| Non-PWID | N | 0.03 | Non-PWID | Intranasal | 0.523 | N | Y | 0.021 |
| Non-PWID | Y | 0.11 | Non- PWID | Intramuscular | 0.621 | Y | Y | 0.001 |

Table A5. Proportion of MOUD distribution (CTDOC)

|  | Incarceration | | Community |
| --- | --- | --- | --- |
| MOUD type | Male | Female |  |
| METH | 1 | 0.7 | 0.3 |
| BUP | 0 | 0.3 | 0.6 |
| NAL | 0 | 0 | 0.05 |
| INJBUP | 0 | 0 | 0.05 |

Table A6. Cost of MOUD; incarceration costs derived from conversations with CT DOC; community methadone from expert opinion

|  | Incarceration | | Community |
| --- | --- | --- | --- |
| MOUD type | Drug cost | Administration cost |  |
| METH | $493 | $7128 | $5173 |
| BUP | $862 | $7176 | $2094(31) |
| NAL | $18472 | $9466 | $6364(31) |
| INJBUP | $34011 | $0 | $19457(32) |
